# The Effect of High Fat Diet on Cerebrovascular Health and Pathology: A Species Comparative Review

**DOI:** 10.3390/molecules26113406

**Published:** 2021-06-04

**Authors:** Benjamin Zimmerman, Payel Kundu, William D. Rooney, Jacob Raber

**Affiliations:** 1 Department of Behavioral Neuroscience, Oregon Health & Science University, Portland, OR 97239, USA; zimmermb@ohsu.edu (B.Z.); kundup@ohsu.edu (P.K.); rooneyw@ohsu.edu (W.D.R.); 2 Advanced Imaging Research Center, Oregon Health & Science University, Portland, OR 97239, USA; 3 Beckman Institute for Advanced Science and Technology, University of Illinois at Urbana-Champaign, Urbana, IL 61801, USA; 4 Departments of Neurology and Radiation Medicine, Division of Neuroscience, ONPRC, Oregon Health & Science University, Portland, OR 97239, USA

**Keywords:** high-fat diet, species differences, cardiovascular health, metabolism, cerebrovasculature, cognition

## Abstract

In both humans and animal models, consumption of a high-saturated-fat diet has been linked to vascular dysfunction and cognitive impairments. Laboratory animals provide excellent models for more invasive high-fat-diet-related research. However, the physiological differences between humans and common animal models in terms of how they react metabolically to high-fat diets need to be considered. Here, we review the factors that may affect the translatability of mechanistic research in animal models, paying special attention to the effects of a high-fat diet on vascular outcomes. We draw attention to the dissociation between metabolic syndrome and dyslipidemia in rodents, unlike the state in humans, where the two commonly occur. We also discuss the differential vulnerability between species to the metabolic and vascular effects of macronutrients in the diet. Findings from animal studies are better interpreted as modeling specific aspects of dysfunction. We conclude that the differences between species provide an opportunity to explore why some species are protected from the detrimental aspects of high-fat-diet-induced dysfunction, and to translate these findings into benefits for human health.

## 1. Introduction

Beginning in the early 1990s, Greenwood and Winocur published some of the first studies describing the deleterious effects of a high-fat diet (HFD) on cognition, using rodent models [1,2]. In young rats, they showed that a diet of 40% fat, compared to the more standard 4.5% fat, for three months, impaired working memory as well as spatial memory [1]. While the largest impairment was in the HFD group fed a lard-based diet high in saturated fatty acids (SFAs), there was also an impairment in the soybean-oil-based, high-polyunsaturated fats (PUFA) diet [1]. This suggests a deleterious role of high-fat diets in cognition, especially for diets high in SFAs. Since that time, in numerous studies, the deleterious effects of a HFD on cognition were revealed, the biological mechanisms of which are beginning to be elucidated [3,4]. In rodents, hippocampal-dependent learning and memory seems especially vulnerable to HFD-induced cognitive impairment [4,5]. 

In human epidemiological studies, there is an association between the consumption of an HFD and cognitive impairment, as well as risk of developing neurodegenerative disease. In the population-based prospective Rotterdam Study, which followed non-demented participants aged 55 and older for an average of 2.1 years, increased consumption of total fat, SFAs and cholesterol were associated with a greater incidence of dementia [6]. This relationship was maintained even after adjustment for total energy intake, suggesting that these nutrients have a specific deleterious effect on the brain, above a simple calorie surplus. Additionally, dementia related to vascular dysfunction was most strongly related to total fat and saturated fat [6]. A diet high in total calories and total fat was associated with higher incidence of Alzheimer’s disease (AD) in those carrying the APOE ϵ4 allele [7]. This is similar to what was found in mice carrying human APOE alleles [8,9]. Interestingly, high PUFA consumption through high fish and nut intake was associated with a lower incidence of dementia and, specifically, AD, in contrast to early findings in rodents, indicating that a diet high in any fat, including primarily PUFAs, is deleterious to cognitive health [1,6,10].

Thus, in both animal models and humans, a high-fat diet has been related to declining cognitive ability, although a high PUFA diet has not been related to declining cognitive abilities in humans. Examples of high-fat foods include pastries, fried foods, and fatty cuts of meat. Examples of low-fat foods include most fruits and vegetables, as well as beans and legumes. From the mostly observational evidence in humans, there is a strong hypothesis that certain types of HFD impair cognition, at least in part, through deleterious effects on the cerebrovasculature. While laboratory animals provide excellent models for more invasive research on the molecular cascades that lead to impaired cerebrovascular function as a consequence of a high-fat diet, important differences in terms of how rodents and primates process dietary fat, and what factors may affect the translatability of mechanistic research in animal models, need to be considered. In this review, we seek to elucidate some of these issues to generate a framework for thinking about the successful translation of results from animal research to interventions in humans.

### 1.1. Environmental Factors in Translating HFD Research

**Figure 1:**
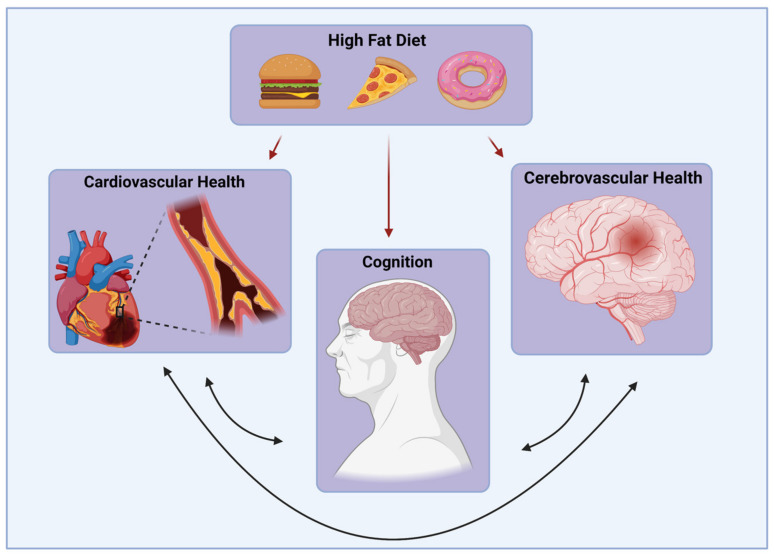
Detrimental Effects of an HFD. Consumption of an HFD can impair cardiovascular, cerebrovascular, and cognitive health.

Animal models, especially rodent models, are often used to study the deleterious effects of chronic HFD consumption (Figure 1).

However, there is a wide translational discrepancy in the mechanisms of impairment and, thus, potential therapies. Species differences might exist in the HFD literature for several reasons. Experimental HFDs often far exceed the upper limit of fat consumption typical of humans consuming a Western diet [11]. While a Western diet has 30–40% of its total energy as fat, obesogenic diets for rodents often contain 60% of total energy as fat. This, in turn, reduces housing and experiment costs and results in animals becoming obese more quickly [11]. However, the metabolic changes achieved by different high-fat diets in rodents vary with fat content. In a study comparing metabolite content in the lung of mice fed an HFD (45% kcal from fat), a very high HFD (60% kcal from fat), or a control diet, 80 metabolites were altered in the lungs of mice fed a diet high in fat compared to standard chow. However, less than half of those 80 altered metabolites were shared between the two HFD groups, indicating altered metabolic responses to a 45% fat diet compared to a 60% fat diet [11,12]. Additionally, in most HFDs used in rodent studies, there is an inverse relationship between fat content and sucrose, with the lowest fat diets containing the most sucrose, the opposite of the pattern seen in human diets [11]. Given the role of sugar consumption in metabolic dysfunction, this is potentially problematic. It should also be noted that nearly all rodent models of HFD use lard as the primary source of fat. Therefore, these studies are not generalizable to diets high in fat generally, but rather diets high in saturated fatty acids from animal sources. Fatty acids from plant sources (e.g., walnuts) could protect against pathology in the aged brain [13]. Additionally, rodents in HFD studies are confined to a small space and, thus, are extremely sedentary, while human activity levels vary.

### 1.2. Metabolic Factors in Translating HFD Research

There might be differences in how mice regulate body weight compared to primates. In a study testing 29 different diets across five common laboratory mouse strains, only increases in dietary fat were found to increase adiposity, not increases in sucrose or protein content [14]. However, in human studies, high-carbohydrate diets easily lead to excess energy intake and weight gain [15]. Additionally, the strain, age and sex of rodents affect how much weight is gained on an HFD, as well as the localized distribution of adipose tissue in the body, which affect health outcomes dramatically [16]. C57BL/6 mice are commonly used in HFD studies, because they are genetically prone to the development of insulin resistance [17].

The non-human primate (NHP) HFD literature recapitulates many main findings from the rodent HFD literature. Rodents and NHPs share similarities in conserved mammalian physiological systems compared to humans, but rodents are much less resource-intensive as a scientific model and have shorter gestation times and lifespans. NHPs are more similar to humans than rodents in several important aspects of metabolic physiology, grey/white matter distribution, cortical folding, and vascular networks [18]. In NHPs and humans, the major site of de novo lipogenesis is the liver, while, in rodents, it is adipose tissue [19]. Primates also have similar classes of serum lipoproteins, pathways of thermogenesis, and insulin-mediated glucose regulation compared to rodents [19].

There might be important differences in how rodents and primates respond to a HFD. Humans do not generally become obese on an HFD unless that diet is also high in carbohydrates [20]. In fact, humans on a high-fat diet that is low in carbohydrates have better cardiovascular and metabolic outcomes than calorie-matched individuals on a high carbohydrate diet [20,21,22]. The benefits to cardiovascular health include reduced triglycerides, higher high-density lipoprotein particles, and lower low-density lipoprotein particles [21]. This is true even if the diet is high in saturated fat and in the absence of weight loss [20,21,23,24,25,26]. However, mice consuming an HFD with zero carbohydrate content more readily become obese compared to mice consuming standard chow, even though the two groups consume a similar number of total calories [20]. These mice develop a metabolic profile similar to that seen in humans consuming a high-carbohydrate diet, including increased fat deposits in the liver and heart, impaired glucose regulation, and insulin resistance [20]. Rodents develop dysfunctional metabolic and cardiovascular outcomes after markedly short periods of HFD consumption, often in just 6–8 weeks [27]. Thus, rodents may be comparatively more vulnerable to metabolic and cardiovascular dysfunction in response to an HFD than humans and this should be considered when evaluating the health effects of an HFD in rodent models. HFD-induced obesity in rodents can be used to model dysfunctional metabolism, and perhaps used to elucidate pathways leading to specific types of dysfunction, but should not be extrapolated to infer the relative health impact of HFD consumption in humans.

A related issue is whether calorie restriction (CR) increases longevity and healthspan in a consistent manner across species. There are numerous studies highlighting the benefits of CR in rodents, NHPs, and humans, as well as other model organisms, making it likely that CR mechanisms found in other species may translate to human health [28,29,30,31]. However, it should be noted that the mechanisms responsible for life extension through CR may differ between model organisms [32]. In addition, some researchers have pointed out that the relative unhealthiness of experimental control groups (e.g., through obesity or metabolic dysfunction) may lead to overestimating the effect of CR itself [29,32].

Another potential species difference in the response to a HFD is the presence or absence of a gallbladder. Interestingly, though very related species, mice possess a gallbladder, while rats do not [33]. In rats, bile from the liver flows directly into the small intestine through the hepatic bile duct [33]. One of the functions of the gallbladder is to store and concentrate bile produced in the liver, which aids in the breakdown of dietary lipids. Thus, in rats, hepatic bile is not concentrated, as it is in mice and in primates. However, at production, rat hepatic bile is eight times more concentrated than mouse hepatic bile, which may compensate for this species difference. In humans without gallbladders, the liver releases bile directly into the small intestine, as there is nowhere for the bile to be concentrated. This allows for the digestion of most foods, but makes the digestion of very high-fat foods difficult [34]. In addition to bile concentration, recent research has uncovered more nuanced and active functions of the gallbladder, including the regulation of bile acid composition and cholesterol [35]. Thus, the presence or absence of a gallbladder in a given animal model may contribute another aspect of variability to the HFD literature.

In humans, it has been known for some time that type II diabetes (T2D), dyslipidemia and obesity are often comorbid and cluster with hypertension and cardiovascular disease, and that the consumption of a HFD is a risk factor for all these diseases [36]. Hypertension, diabetes, and hyperlipidemia have long been recognized risk factors for stroke and cardiovascular disease and are now recognized risk factors for vascular cognitive impairment and many neurodegenerative conditions [37,38]. Vascular abnormalities leading to cognitive impairments are responsible for 20% of dementia cases [39], although impaired vasculature may play a role in even more cases of dementia. Cerebrovascular impairment includes atherosclerosis, arteriolosclerosis, thrombus, embolus, and microbleeds [39]. Vascular impairment plays a role in age-related cognitive decline, as well as in neurodegeneration [40]. Cerebral hypoperfusion is a major contributor to cognitive decline [41,42]. Several rodent models were generated to study the effects of chronic hypoperfusion on the development of vascular cognitive impairment and dementia [43,44,45,46], although it is important to bear in mind that sudden, experimentally induced arterial stenosis or ischemic conditions may not perfectly mimic the damage resulting from physiological adaptations to more gradual changes in arterial pulsatility or hypoperfusion. 

In a few studies, the direct effects of an HFD on changes in cerebrovasculature were investigated. Most studies to date have focused on direct HFD effects on the peripheral vasculature, or indirect effects on peripheral vasculature through systemic diseases related to HFD consumption, such as metabolic syndrome. In a hamster model of diet-induced hyperlipidemia, changes in the brain microvasculature were observed, including irregularly shaped vessels with large perivascular spaces and enlarged endothelial cells [47]. In rats with diet-induced metabolic syndrome, cerebral microvascular blood flow was significantly worse after experimentally induced ischemia compared to rats fed a control diet [48]. Thus, metabolic syndrome exposed the brain to higher states of hypoxia due to impaired cerebrovasculature.

Sex differences in obesity and metabolic function must be considered. However, there are species differences in how sex differences manifest. Male mice are more prone to diet-induced obesity than female mice, while, in rats, this sex difference is less pronounced [49,50,51,52,53,54,55]. For insulin resistance and glucose intolerance, male mice and rats are more susceptible than female mice and rats [52,55,56,57,58,59]. For example, male Zucker Diabetic Fatty rats develop severe dysregulation of glucose and insulin on a normal chow diet by young adulthood, while females of the same strain maintain normal levels, despite becoming obese at a similar degree to males [52]. In humans, men suffer from insulin resistance more than women and develop diabetes at a lower body mass index, despite women having higher rates of obesity than men [52,60,61,62,63]. The incidence of T2D overall is also higher in men than women [52,64]. However, women with metabolic syndrome are at higher risk of developing cardiovascular risk (4-fold) than men with the syndrome (2-fold) [36]. Obesity is an independent predictor of cardiovascular disease, especially in women [36,65,66]. These sex differences are partly due to the effects of sex hormones on fat distributions. Androgens lead to a higher deposition of visceral and central adiposity, while estrogens tend to lead to subcutaneous fat, which is not as metabolically detrimental [52]. Thus, these species-specific sex differences must be considered during the analysis of studies on HFD. However, it is important to be very careful when extrapolating sex differences in the animal model to sex differences in humans. Rodents and humans share the trait of males being more susceptible to the deleterious effects of diet-induced obesity, as well as the sex-hormone-mediated patterns of fat deposition, but not the consistent susceptibility to diet-induced obesity in one sex over another.

With these broad species differences in mind, we will now review the findings in the rodent, NHP, and human literature on the effects of an HFD on the cerebrovasculature. We will highlight species similarities, as well as differences, to better understand which model organisms are most suitable to address specific questions in this field.

## 2. Effects of HFD on the Cerebrovasculature

### 2.1. Cerebral Arterial Stiffening and Inflammation

The downstream consequences of an HFD, including diabetes mellitus, hypertension, dyslipidemia and obesity, have long been recognized as predictors of cardiovascular disease and arterial stiffening in humans. Besides increased risk of heart attacks and stroke, arterial stiffening might cause downstream damage to the brain through increased pulsatility [67,68]. Using non-invasive optical methods in humans, the stiffness of cerebral arteries can be inferred through the shape of the pulse wave [69,70]. In recent years, investigations of parameters of the cerebral arteries have revealed that cerebral arterial stiffness in humans varies by region and predicts cortical thickness and volume, white matter lesions, and cognition [69,70,71,72].

The mechanisms leading to cerebral arterial stiffening and the downstream microvascular consequences of increased pulsatility have begun to be elucidated through studies primarily involving rodents. Thus, a critically important question is whether the inflammatory mechanisms leading to arterial stiffening and damage to the microvasculature are similar across mammalian species.

Mouse models are by far the most commonly used mechanisms to investigate arterial stiffening on cerebral microvascular functioning. They are good, but not perfect, mechanistic models for cerebral microvascular functioning in humans. For one, mice show similar patterns of advancing arterial stiffness with age, as do humans [73]. Additionally, like humans, an HFD increases arterial stiffening relative to mice fed normal chow [73]. However, rodent models are generally limited by major differences in the subsequent pathology following arterial stiffening and in molecular factors that greatly differentiate risk. There is increased natural arterial stiffening in aging rodents, which seems to be accelerated by the consumption of an HFD [73]. However, unlike humans, wild-type rodents do not naturally develop cardiovascular or neurodegenerative diseases, due to differences in lipid metabolism and amyloid-β production [74]. Thus, while genetic manipulations or surgical interventions have been widely used to better mimic the damaging effects of arterial stiffening in humans, these interventions must be considered carefully, since they often substantially vary from the natural process in humans. Most concerningly, experimental manipulations that cause sudden acute changes may fail to model the long-term adaptive processes that occur in humans [75].

Experimental manipulations to examine the effect of arterial stiffening on the subsequent molecular and physiological effects on the brain have been extensively reviewed recently by Winder et al. [74]. Some of these models involve genetic manipulations to induce arterial stiffening by reducing elastin or mutating fibrillin, which may be confounded by effects on the development and undesired susceptibility to side-effects in other tissues [74,76,77,78]. Other models involve manipulations that cause sudden increases in stiffness or pulse pressure by surgically constricting large arteries or calcifying the carotid artery [79,80,81,82,83,84]. The limitation of these types of models is the mismatch between sudden, and acute increases in blood pressure compared to the gradual process of stiffening in human aging.

The existing research involving animal models has provided excellent supporting evidence that increasing pulse pressure can cause downstream inflammation and damage to the cerebral microvasculature, with negative consequences for cognition [74]. However, it is important to note the limitations of the existing models in building testable hypotheses about potential treatments. Since many of the models involve acute manipulations to arterial pressure, it is natural to experiment primarily with the reduction in pressure, rather than intervening in the slow mechanisms that cause stiffening over long periods of time. This limitation is exemplified in the current treatment of hypertension. Many available medications for hypertension work well for reducing blood pressure, but do not treat the factors causing hypertension to appear, and there is surprisingly scant evidence that hypertension treatment improves cognitive performance [85,86].

In other recent studies, the appropriateness of using mice as models of atherosclerosis using genetic analysis has been studied. Although there is still not a consensus on the use of mouse models for all pathways leading to arterial dysfunction in humans, there is support for using mouse models for studies on genetic differences related to lipoprotein metabolism as a predictor of arterial disease [87,88]. However, this is confounded by the complexity introduced by the overall differences between humans and rodents in lipid metabolism [89]. Another interesting difference between the progression of arterial stiffness in humans and other mammals is in the structure of the abdominal aorta [90]. The human abdominal aorta has fewer layers relative to its diameter compared to other species, resulting in elevated mean tension, which may interact with the pressure throughout the vascular system in complex ways.

An HFD seems to impact arterial stiffness primarily through the complex association of metabolic dysfunctions, including obesity, hypertension, insulin resistance, metabolic dyslipidemia, impaired renal function, and increased inflammation (extensively reviewed by Aroor et al. [91]). Arterial stiffness is driven by the reaction of vascular cellular components to endocrine factors, cytokines, immune cells, and signaling from perivascular adipose tissue. These interactions lead to impaired endothelial nitric oxide through multiple mechanisms, which, in turn, promote vascular fibrosis. At the same time, maladaptive inflammatory responses lead to additional endothelial dysfunction.

Overall, the interventions available in mice to induce downstream effects of arterial stiffening on the brain are very promising avenues of mechanistic research. However, in future research, more gradual stiffening models are required to determine if there are physiological differences that may manifest as adaptations to a slower process of stiffening.

### 2.2. Blood Brain Barrier

**Figure 2:**
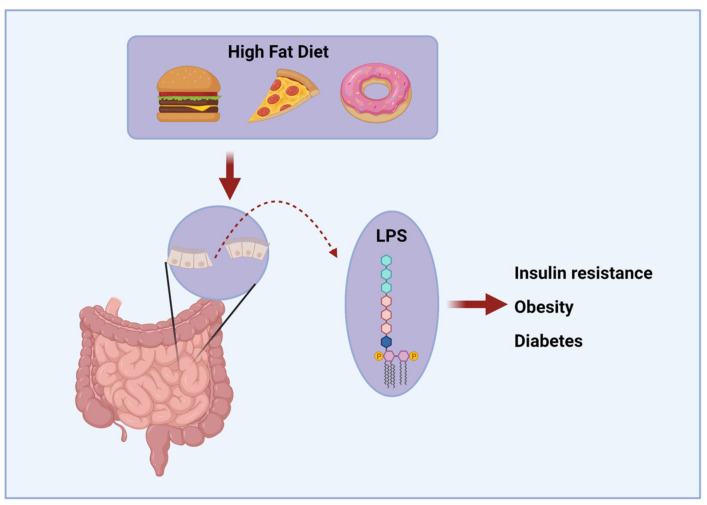
HFD effects on BBB integrity. A diet high in saturated fatty acids can impair BBB integrity and impair cognition.

In the blood vessels that vascularize the central nervous system (CNS), a system of endothelial tight junctions forms a selective barrier controlling the movement of ions, molecules, and cells between blood and CNS parenchyma (Figure 2).

The integrity of the blood–brain barrier (BBB) is essential for brain volume/pressure regulation, metabolic homeostasis, and BBB dysfunction has been implicated in a wide range of neurological disorders, including multiple sclerosis, stroke, epilepsy and AD [92,93]. The common underlying mechanism likely involves an environment of prolonged and chronic inflammation, which may result, in part, from chronic consumption of an HFD, such as a Western diet [94]. Long-term consumption of an HFD may lead to leakage in the intestinal barrier [93,95]. This leads to the increased translocation of gut bacteria to the blood, and circulation of lipopolysaccharides (LPS) found in the outer membrane of Gram-negative bacteria. LPS binds to Toll-like receptor 4 (TLR4), which leads to the activation of NF-κB, which regulates the transcription of genes related to innate immunity and inflammatory responses [96]. Elevated LPS may even be a triggering factor for the development of insulin resistance, obesity, and diabetes that often results from chronic HFD consumption. Mice that were continuously infused with LPS for 4 weeks developed impaired fasting glucose, insulinemia, as well as increased whole-body and liver fat to a similar extent as was seen in HFD-fed mice [97]. In addition, the hypertrophy of adipocytes leads to inadequate oxygenation of visceral adipose tissue, which also plays a role in the link between obesity and inflammation [98]. Adipose inflammation typically develops in white adipose tissue, but this also impairs the ability of brown adipose tissue to take up and utilize glucose, thus further impairing metabolic health [99].

**Figure 3:**
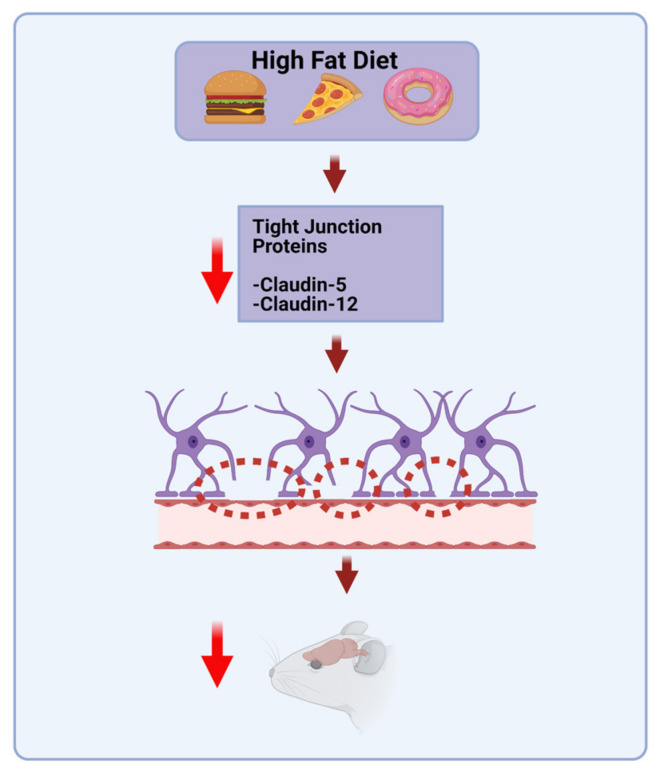
HFD diet can induce inflammation. Chronic HFD consumption can lead to leakage in the intestinal barrier, leading to elevated serum levels of LPS. Elevated LPS may be a triggering factor for the development of insulin resistance, obesity, and diabetes.

Diet can play a role in BBB integrity (Figure 3).

A diet high in SFAs and glucose selectively impairs hippocampal learning and memory as well as the integrity of the BBB in rats [5]. Specifically, there was a reduction in mRNA expression of the tight-junction proteins Claudin-5 and -12 in the choroid plexus and the BBB. Additionally, there was increased blood-to-brain permeability of an exogenously administered dye in the hippocampus, but not in the prefrontal cortex (PFC) or striatum, suggesting that the hippocampus is especially vulnerable to HFD-induced disruption of the BBB [5]. Similarly, in rats fed a diet high in SFAs and cholesterol, a reduction in endothelial barrier proteins suggested an increased permeability of the BBB in the Cornu Ammonis (CA1) region of the hippocampus as well as the parietal cortex, compared to rats fed standard chow [100]. Maternal HFD can even lead to impaired BBB integrity in the offspring. In diet-induced mouse dams, neonatal offspring were found to have a more permeable BBB around the arcuate nucleus of the hypothalamus, an area associated with regulation of body weight [101].

The HFD-induced disruption of the BBB in rodents might be mediated by individual propensity to gain adiposity from an HFD, suggesting that BBB disruption might be secondary to body weight gain [102]. The HFD-induced disruption of the BBB might also allow for the increased migration of peripheral immune cells into the CNS [93]. There is some evidence that BBB disruption might be reversible. In mice consuming an HFD, supplementation with reservetrol attenuates the disruption through actions on occludin and ZO-1 tight junctions [103]. There is also an interaction of HFD consumption and aging on BBB integrity. In a comparison of young vs. aged diet-induced obese mice, aging exacerbated obesity-induced systemic inflammation and BBB disruption [104]. Cholesterol rich diets also seem to damage the BBB. Rabbits fed a 1% cholesterol diet for seven months showed BBB disruption, as well as increased amyloid beta and iron deposition, compared to rabbits fed normal chow [105].

In NHPs and humans, there are far fewer studies on the effects of a HFD on the BBB. Reduced BBB integrity plays a role in vascular dementia and increases with aging, even in healthy humans [39,106]. Thus, if an HFD contributes to impaired BBB integrity, this might worsen age-related as well as pathological cognitive impairment.

There is some evidence that chronic consumption of an HFD changes the BBB’s permeability to some molecules via modulation of active transport mechanisms. Insulin enters the brain from the peripheral circulation via active transport [107,108]. In the brain, unlike its functions in the periphery, insulin acts to regulate feeding and cognition through mechanisms largely independent of glucose utilization [107]. Insulin transport across the BBB seems to be altered with obesity and peripheral insulin resistance in humans. In lean humans, an infusion of peripheral insulin increased both stimulated and spontaneous cerebrocortical activity [109]. This effect was not seen in obese individuals [109]. When measured directly via lumbar puncture, peripheral insulin resistance leads to the transport of a lower amount of insulin across the BBB [108]. The active transport of glucose across the BBB was similar between lean and obese individuals, suggesting that peripheral insulin resistance can lead to hyperglycemia in the brain [108]. The reduced transport of insulin across the BBB has also been observed in obese animals of various other species, including rodents [110].

### 2.3. Microvascular Rarefaction and Other Microvascular Changes

How an HFD affects morphological features of the cerebral microvasculature is still being explored. In most current models, multiple mechanisms are identified to explain how HFD and associated dysfunction affects the microvasculature, which strongly overlap with the mechanisms of endothelial dysfunction in the larger arteries. In addition, there seems to be evidence for a cascade of effects that lead to microvascular changes due to increased pulsatility from arterial stiffening, as discussed earlier in this review.

In the few existing reports in which the effect of diet on microvascular morphology was investigated, reduced microvascular density in the brain, as well as dysfunctional remodeling of the endothelial surface that leads to reduced blood flow and an increased platelet aggregation, are observed. The cortical cerebral microvasculature of Wistar rats fed an HFD showed a thickening of the vascular basal laminae as well as increased microvilli and indentations on the endothelial surface [111]. They also had microvascular rarefaction compared to rats fed normal chow. In another study, the effects of a high-fat, high-sugar diet on microvascular morphology in middle-aged rhesus macaques was investigated. Exposure to two years on this diet resulted in the dysregulation of endothelial nitric oxide synthase and reduced capillary density in the cerebral cortex [112].

Future efforts are warranted to confirm these observations and to determine whether the molecular mechanisms leading to similar morphological changes in the cerebral microvasculature observed in rats and primates to date are identical.

### 2.4. Systemic Factors

Other factors related to HFD are also predictors of cerebrovascular disease, including aging, hypertension, elevated cholesterol, diabetes, obesity, and atherosclerosis [39]. In addition, there may be complex interactions with behavior that moderate some of the effects of HFD, such as stress and inactivity [113,114,115,116]. Obesity is associated with the development of many systemic diseases, including diabetes mellitus, hypertension, dyslipidemia, as well as ischemic heart disease [117]. All of these factors can interact to produce dysfunctional metabolic signaling, which directly plays a role in inflammation and subsequent vascular impairment.

These conditions can lead to cerebral hypoperfusion and increase the likelihood of developing cognitive impairment [48,118]. In obese Zucker rats, obesity led to worse stroke outcomes, measured by infarct size, and remodeling of the middle cerebral artery compared to lean control rats [119,120]. These adverse outcomes were partially prevented through the management of hypertension via the administration of hydrochlorothiazide [119,120].

In rodents, an HFD reliably leads to poor cardiovascular outcomes, including hypertension. Rats fed an HFD for 8 weeks developed higher systolic blood pressure, an increase in inward Ca^2+^ current density, and higher serum fatty acids [27]. Obese Zucker rats developed metabolic syndrome and a substantial reduction in microvascular density in the cerebral cortex, beginning in young adulthood and progressively worsening with age, as well as other organ systems, including myocardium, kidney, and skeletal muscle [121]. Interestingly, this microvascular rarefaction did not seem to be a direct adaptation to chronically elevated perfusion pressure, as is often thought, because treatment with captopril (an angiotensin converting enzyme inhibitor) or hydralazine (a systemic vasodilator) was equally effective at alleviating hypertension, but had disparate effects on cerebrovascular rarefaction [121]. Captopril was significantly more effective at preventing a loss of cerebral microvascular density. Captopril treatment also leads to improved glycemic control, which seems to be a more important predictor than hypertension of microvascular rarefaction in rodents [121,122]. Indeed, treatment with both metformin and rosiglitazone, both of which improve insulin sensitivity and glycemic control, also improved cerebral microvascular density. However, as mentioned earlier, mice do not spontaneously develop atherosclerosis [19,123]. Atherosclerosis is a chronic inflammatory disorder, affecting medium and large arteries, which underlies most cardiovascular disease [123]. The condition involves both the cells comprising blood vessel walls and the immune system, which is activated in response to hyperlipidemia [123]. Although mice do not develop this spontaneously, they remain the most popular model to study this condition [123]. The most common models are mice with genetic deletion of *Apolipoprotein E* or the low-density lipoprotein (LDL) receptor [8,123]. NHPs, on the other hand, do spontaneously develop atherosclerosis [19].

The effects of high-fat food on endothelial function can manifest after just a single meal, suggesting immediate signaling differences in response to HFD rather than just chronic alterations to metabolic function [124]. In healthy human volunteers, flow-mediated vasoactivity decreased in those fed a high-fat meal compared to an isocalorically matched low-fat group in the postprandial 6 h [124]. Similar changes have been found after 90 days of high-fat diet consumption [125]. Endothelial function in the 90-day study was assessed after a 12-hour fast, indicating that the results are not associated with a transient postprandial state, but stable physiological changes [125]. The mechanism through which a high-fat diet interferes with endothelial function likely involves the free-radical inactivation of nitric oxide [125,126]. High fat consumption increases mitochondrial β-oxidation of free fatty acids, ultimately resulting in the increased production of reactive oxygen species [127]. Indeed, oral ingestion of antioxidants partially protects against HFD-induced endothelial dysfunction [125,128,129].

An HFD can also affect the gut microbiome. In mice, switching to an HFD was associated with a decrease in *Bacteroidetes* and an increase in both *Firmicutes* and *Proteobacteria* species [130]. Changes to the gut microbiome seem to be driven by the HFD itself, not obesity [130,131]. Germ-free mice are resistant to obesity when fed a high-fat and high-sugar diet, which typically makes conventional mice obese [132]. In germ-free mice, phosphorylated AMP-activated kinase (pAMPK) was increased in the liver and skeletal muscle, as well as the downstream targets of pAMPK, leading to increased host metabolism of fatty acids [132]. The germ-free mice also showed increased expression of fasting-induced adipocyte protein, a lipoprotein lipase inhibitor, which led to increased mitochondrial fatty acid oxidation [132].

In humans, obese individuals have a lower relative abundance of *Bacteroidetes* and higher relative abundance of *Firmicutes* species compared to lean controls [133]. This can be modulated by eating an energy-restricted diet, with increases in *Bacteroidetes* species and decreases in *Firmicutes* species that correspond to the degree of weight loss [133]. Overall, the alpha diversity of the gut microbiome was not different between obese and lean individuals [133].

The gut microbiome plays a role in cardiovascular health, both positive and negative, through several recently emerging mechanisms. This typically occurs in the form of bacterial metabolites. Metabolic products made by gut bacteria from ingested food can have a beneficial impact on cardiovascular health. Butyric acid, degraded from dietary fiber, can inhibit cholesterol absorption and prevent atherosclerosis [134,135]. Anthocyanins have been shown to have anti-atherogenic effects, but the compound itself is absorbed very poorly [136]. It is likely that the bacterial metabolites of anthocyanins are exerting the beneficial effects. One such metabolite is protocatechuic acid, and was shown to attenuate atherosclerosis in a mouse model [136]. Two microbial metabolites in particular, trimethylamine-N-oxide (TMAO) and short-chain fatty acids (SCFAs), play a large role in cardiovascular risk [137]. High levels of TMAO increase cardiovascular risk in humans, likely through the regulation of cholesterol metabolism and oxidative stress, even when traditional risk factors are controlled for [138,139]. High levels of SCFAs, a microbial product of dietary fiber consumption, are associated with better cardiovascular outcomes in humans [137]. At the phyla level, the human and mouse microbiota are similar. In both species, Firmicutes and Bacteroidetes comprise more than 90% of the gut bacteria [133]. However, in humans, the gut microbiome consists of more Firmicutes and fewer Bacteriodetes compared to mice [140]. Additionally, there are nine genera of uniquely human gut microbes that are not commonly present in mice [140]. However, despite these species differences, human and mouse microbiomes share representatives of the same phyla as well as a substantial fraction of common genera, supporting the use of mice to study microbiome effects on health, including cardiovascular health [140].

Diabetes is associated with cardiovascular dysfunction. Cardiovascular disease is the leading cause of morbidity among patients with T2D [36,141]. In humans, T2D is a complex disorder characterized by progressively worsening insulin resistance and pancreatic β-cell dysfunction [142]. In humans, pancreatic β-cells adapt to insulin resistance initially by increasing in both size and number [143]. As hyperglycemia and elevated free fatty acids persist, an increase in reactive oxygen species (ROS), increases in intracellular calcium, and endoplasmic reticulum-related stress all contribute to the impairment of insulin secretion and, ultimately, apoptosis in β-cells [143]. However, this is not necessarily the case in rodents. While rodents often become obese on a diet of 40–60% calories from fat and develop insulin resistance, many do not readily develop beta cell failure [142]. Instead, they have a markedly increased production in the number of β-cells [142]. Because of this, many rodent strains are slower to develop fasting hyperglycemia after the onset of obesity than humans, and many strains do not reach the upper end of fasting hyperglycemia seen in human diabetes [52,144]. Additionally, they develop more robust hyperinsulinemia compared to human patients with diabetes [144]. The C57BL/6J mouse strain, very commonly used as a model of diet-induced obesity due to their propensity to become obese on a high-energy diet, seldom develop frank hyperglycemia at all [52]. To recapitulate β-cell dysfunction in rodents, a β-cell toxin such as streptozotocin is commonly used [144]. However, this is not true of all rodent strains. Some strains and transgenic mouse models, such as apoE-deficient C57BL/6 mice or NOD/ShiLtJ mice, do develop hyperglycemia and recapitulate some aspects of disease [145,146]. Thus, it is important to consider the specific aspects of the human disease that the distinct strains model. For example, in humans, hyperinsulinemia is an independent risk factor for cardiovascular disease [147]. Thus, it is important to know the degree to which a particular model develops more robust insulinemia, rather than frank hyperglycemia.

Several aspects of metabolic syndrome independently lead to cardiovascular injury. Some of these are modeled well by rodents; some are not modeled well by most rodent strains, but may be mimicked in certain strains or in transgenic models. Hypertriglyceridaemia, or triglyceride-rich, very-low-density lipoproteins play a direct role in vascular injury in humans [36]. They are vulnerable to oxidation and are a factor in the early stages of atherosclerosis [36]. High-serum cholesterol is even more strongly associated with cardiovascular risk, as are hyperglycemia and hyperinsulinemia.

Dyslipidemia is one of the strongest predisposing factors for cardiovascular disease [148]. It is characterized by high levels of serum cholesterol, especially LDLs [148]. There are species differences in both normal and pathological lipid profiles. One important species difference is the lack of a cholesteryl ester transport protein, an enzyme involved in plasma cholesterol transport, in mice and rats [148]. This leads to a high HDL-c to LDL-c ratio in rodents, making them less susceptible to cardiovascular disease [149]. NHPs, on the other hand, have a more human-like pattern, with a predominance of non-HDL lipoproteins [148]. Thus, mouse models of atherosclerosis, like those with genetic deletion of ApoE or the LDL receptor, have been successfully used to elucidate mechanisms of action in human atherosclerosis, but many mouse models of metabolic syndrome and diabetes, such as the Zucker diabetic fatty rat, do not show similar profiles of dyslipidemia to humans and do not develop atherosclerosis [148]. Thus, the important effects of HFD consumption and metabolic syndrome on the vasculature are not recapitulated in these models.

Finally, there is species and strain heterogeneity in the vulnerability to diet-induced obesity. Rodent strains, for example, are differentially prone to becoming obese when exposed to an HFD [150]. Sprague Dawley rats bred for their resistance to weight gain on a high-fat, high-sucrose diet showed similar weight gain over 10 weeks on this high-energy diet, by reducing their intake, as rats fed standard chow [151]. When both obesity-prone and obesity-resistant rats were fed a standard chow diet, obesity-prone rats had 59% higher neuropeptide Y (NPY) mRNA expression in the hypothalamus, a known stimulator of food intake, than obesity-resistant rats [152]. When all rats were fed a high-energy diet for 14 weeks, NPY mRNA expression was reduced dramatically in obesity-prone rats and remained well below the level of obesity-resistant rats, made similarly obese on a highly palatable diet [152]. When obese-obesity prone rats were food-restricted to reduce body weight, NPY levels increased and remained high, indicating that NPY levels in these rats are not subject to metabolic regulation, but operate under a genetic set point [152]. Thus, NPY levels in the hypothalamus are an important genetic determinant of an individual’s vulnerability to diet-induced obesity.

### 2.5. Maternal HFD Effects on Offspring

Evidence from animal models, as well as human epidemiological studies, indicate that the intrauterine environment plays a role in the metabolic health of the offspring [153]. The dependency of fetal development on maternal nutrients leaves the fetus vulnerable to early-life metabolic programming, which can exert effects throughout the lifespan of the offspring. Across animal models, maternal undernutrition leads to dysfunction of the insulin growth factor axis and, thus, poor fetal growth, as well as the development of metabolic disease later in life [154]. The effects of maternal overnutrition were more recently investigated, as this reflects the typical pattern of consumption in the Western world. Across rodent and primate models, maternal HFD also predisposes offspring to the development of metabolic syndrome later in life [155]. These changes are thought to occur through epigenetic programming of the offspring in utero as well as early in life. Early-onset obesity is closely associated with cardiovascular disease later in life [156,157,158,159]. One possible mechanism of the adverse effects of a maternal HFD on offspring is the lack of white adipose tissue early in development. In most mammalian species, including rodents and primates, white adipose tissue does not appear until the third trimester [160,161]. White adipose tissue is the main repository of excess lipids, and without it, systemic insulin resistance, dyslipidemia and fatty liver occur in rodents, non-human primates, and humans [162,163,164,165,166].

In mice and rats, a long-term as well as a short-term maternal HFD leads to hypertension in the offspring later in life [167,168]. This is true even if pups are fed a balanced diet from weaning onwards, suggesting the strong role of in utero epigenetic programming [169]. Similarly, in mice genetically predisposed to T2D, maternal HFD led to increased systolic blood pressure in offspring as early as 13 weeks of age, which is considered young adulthood for mice [170]. Thickening of the left ventricular wall, a predisposing factor for congestive heart failure, was also found in the offspring of rat dams fed an HFD [168]. In Sprague–Dawley rats, a lard-rich maternal diet led to various adverse metabolic outcomes in adult offspring, including hypertension, insulin resistance, dyslipidemia, obesity, and endothelial dysfunction in mesenteric arteries [171]. These adult offspring also showed an increase in aortic stiffness and reduced endothelium-dependent relaxation [171]. When kidney function was examined, these rats had reduced renin and Na+,K+-ATPase activity, a probable underlying contributor to the observed hypertension [171]. Interestingly, it may be that offspring exposure to an HFD during gestation offers an adaptive benefit in terms of endothelial dysfunction if those offspring suckle from an HFD-fed dam [169], since offspring exposed from gestation rather than from later, through suckling, had better cardiovascular outcomes.

In some rodent studies, sex-specific effects on offspring of a maternal HFD were reported. In the female offspring of rat dams fed a diet rich in lard, systolic and diastolic pressure was increased at 180 days and abnormal aortic fatty acid composition was also observed [172]. Female offspring developed hypertension even in cases where they were only suckled by, not gestated within, a female consuming an HFD [169]. These effects were not seen in males [172]. However, in both sexes, acetylcholine-induced relaxation in mesenteric arteries, a measure of endothelial dysfunction, was blunted [172]. Together, this suggests that female offspring are more vulnerable to the deleterious cardiovascular effects of a maternal HFD. 

However, a small and transient effect on offspring metabolic and cardiovascular parameters after maternal HFD has been previously reported [173]. In this case, though the dams were on a 60% fat diet for a prolonged period prior to mating, they did not become obese. Instead, after a short period of hyperphagia, they maintained a similar caloric intake to the standard chow group [173]. This suggests that the programming effects of a maternal HFD on offspring metabolic and cardiovascular parameters are a direct result of maternal obesity, not the consumption of a diet high in fat per se.

However, this might not be the case in primates. In an NHP model of maternal HFD, regardless of whether the mothers became obese, the offspring showed a 3-fold increase in liver triglycerides, evidence of hepatic oxidative stress, and a 2-fold increase in percent body fat, at 6 months of age [166].

In NHPs, maternal HFD as well as post-weaning HFD adversely affects vascular health. A maternal HFD through gestation and weaning and subsequent HFD consumption by the offspring led to vascular dysfunction in the juvenile offspring in the form of blunted endothelium-dependent vasodilatation in the abdominal aorta, thickened intima wall and other predisposing factors to early-onset atherogenesis [156]. These changes occurred before the onset of obesity in the offspring, suggesting adverse developmental programming from the maternal HFD, independent of offspring weight [156]. Offspring that were switched to a control diet after weaning were protected from some of these adverse effects.

Therefore, developmental programming from maternal consumption of a HFD leads to blunted vasodilation in both NHPs and rodents [156,171,174]. It should be noted that rodent pups are born at an earlier stage of brain maturation than primate offspring [175]. Thus, modeling in utero HFD exposure in rodents should include extending the period of exposure into lactation.

## 3. Synthesis: Species Considerations for Assessing the Effects of HFD in Preclinical Models

**Figure 4:**
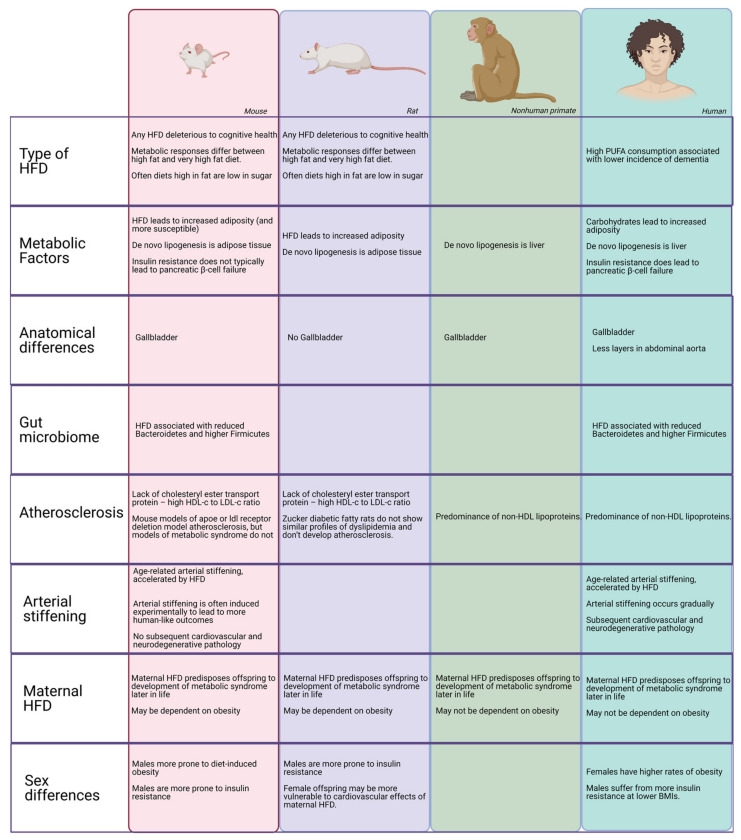
Species differences in HFD response. A representation of major species differences in the effects of HFD that may affect the cerebrovasculature.

Throughout this review, we described species differences in the effect of HFD on cerebrovascular health (for a summary, see Figure 4). These differences can be summarized in broad patterns that may help when interpreting research on an HFD and assessing its translatability to humans.

First, in humans, metabolic syndrome and dyslipidemia often co-occur and may interact when affecting subsequent cerebrovascular dysfunction. This is often not true in rodent models. Thus, a rodent model represents a very specific aspect of human pathology. Experimental manipulations to create a model of some specific aspect of human disease may have “off-target” effects in other areas of the animal’s physiology that need to be considered.

Secondly, the specific models determine which questions can be best asked using them For example, it is common to use procedures to create conditions of great arterial pulsatility or hypoperfusion to examine their downstream effects on the brain. Mechanistically, this research is very useful, but may not accurately model the gradual changes in arterial stiffening and inflammation that lead to downstream effects in humans. This issue is particularly relevant when assessing changes that occur over long durations in humans. Even when a disease process is well-modeled, it is not always clear what constitutes a “chronic” or “long-term” exposure or condition, and whether those time periods are relative depending on the species or absolute.

Third, there are major differences in how rodent models and primates respond to an HFD. Different percentages of calories due to fat result in different effects. It seems that rodents respond to PUFAs in a negative way compared to humans; they are more susceptible in general to HFD-induced obesity than humans, and are more affected by HFD alone than they are by a diet high in carbohydrates, typical of the Western-style diet. Therefore, it is important to identify the specific type of HFD-related dysfunction that models human pathology, and to not make general claims about the relative health impact of HFD consumption based on only rodent studies.

Finally, in both animal models and in humans, there are sex differences in terms of susceptibility to HFD. However, those sex differences are species-dependent. Across animal models, males are more prone to insulin resistance than females. However, the effect of HFD on obesity varies across species, as well as the sex effects of maternal HFD. Thus, it is important to analyze sex differences as potential covariates when investigating the mechanisms of HFD on pathology, but care should be taken to not automatically extrapolate sex differences in different mammalian species to humans.

At the same time, HFD and obesity affect the microbiome of rodents and humans in similar ways. In addition, the effects of maternal HFD seem to be consistent across mammalian species.

It should be noted that modeling the aspects of a condition rather than the complete complicated condition is also done for other preclinical models, including models of neurodegenerative disease. Thus, the HFD literature is not unique in this regard. The apparent overall differences in the effects of HFD across different mammalian species present an opportunity rather than a problem. On one hand, these differences limit, to some extent, the translational value of the mechanistic knowledge that we gain from these animal models, as many of these models are protected from some of the negative effects of HFD on humans. However, fully understanding the mechanisms underlying those relative protections provide novel insight and clinical targets for improving human health.
